# Quadruplex-forming sequences occupy discrete regions inside plant LTR retrotransposons

**DOI:** 10.1093/nar/gkt893

**Published:** 2013-10-06

**Authors:** Matej Lexa, Eduard Kejnovský, Pavlína Šteflová, Helena Konvalinová, Michaela Vorlíčková, Boris Vyskot

**Affiliations:** ^1^Department of Information Technologies, Faculty of Informatics, Masaryk University, Botanicka 68a, 60200 Brno, Czech Republic, ^2^Laboratory of Genome Dynamics, CEITEC - Central European Institute of Technology, Masaryk University, Zerotinovo nam 9, 60177 Brno, Czech Republic, ^3^Department of Plant Developmental Genetics, Institute of Biophysics ASCR, Kralovopolska 135, 61265 Brno, Czech Republic, ^4^Laboratory of CD Spectroscopy, CEITEC - Central European Institute of Technology, Masaryk University, Zerotinovo nam 9, 60177 Brno, Czech Republic and ^5^Department of CD Spectroscopy of Nucleic Acids, Institute of Biophysics ASCR, Kralovopolska 135, 61265 Brno, Czech Republic

## Abstract

Retrotransposons with long terminal repeats (LTR) form a significant proportion of eukaryotic genomes, especially in plants. They have *gag* and *pol* genes and several regulatory regions necessary for transcription and reverse transcription. We searched for potential quadruplex-forming sequences (PQSs) and potential triplex-forming sequences (PTSs) in 18 377 full-length LTR retrotransposons collected from 21 plant species. We found that PQSs were often located in LTRs, both upstream and downstream of promoters from which the whole retrotransposon is transcribed. Upstream-located guanine PQSs were dominant in the minus DNA strand, whereas downstream-located guanine PQSs prevailed in the plus strand, indicating their role both at transcriptional and post-transcriptional levels. Our circular dichroism spectroscopy measurements confirmed that these PQSs readily adopted guanine quadruplex structures—some of them were paralell-stranded, while others were anti-parallel-stranded. The PQS often formed doublets at a mutual distance of up to 400 bp. PTSs were most abundant in 3′UTR (but were also present in 5′UTR). We discuss the potential role of quadruplexes and triplexes as the regulators of various processes participating in LTR retrotransposon life cycle and as potential recombination sites during post-insertional retrotransposon-based genome rearrangements.

## INTRODUCTION

Transposable elements form a significant proportion of eukaryotic genomes, representing ∼50% of the human genome and up to 90% of genomes in some plant species. Long terminal repeat (LTR) retrotransposons that are especially common in plants have a duplicative mode of spreading via RNA intermediate and contain *gag* and *pol* genes [for a review, see (1)]. Retroviruses and some LTR retrotransposons also have an *env* gene, which is necessary for their virulence. In addition to their genes, LTR retrotransposons have many regulatory sequences, such as LTRs containing promoters where transcription of the whole element starts, primer-binding site (PBS) and polypurine tract (PPT) where reverse transcription of the first and the second strands, respectively, starts (2). Retroviruses have additional signal sequences, e.g. a packaging signal sequence (*psi*), located downstream from the PBS site, that allows packaging of RNA into viral cores (3). *Psi* element is a structural feature composed of four stem loop sequences called SL1 to SL4 (4). Stem loop 1 (SL1) is known as primary dimerization initiation site that initiates dimerization of two RNA genomes inside virus particles by formation of a kissing-loop complex (5) facilitating recombination (6). Stem loop sequences are characterized by the formation of hairpin structures indicating that secondary DNA structures are functionally involved in the retrovirus life cycle.

DNA is a conformationally flexible molecule that can adopt not only canonical B- and A-forms but also many other conformations, such as left-handed Z-form, triplex and quadruplex structures [reviewed in (7)]. Unusual DNA conformations are often adopted by regulatory regions where they may play the role of switches in various molecular processes and human diseases (8). Quadruplexes are formed by G-rich sequences in eukaryotic telomeres, G-rich satellites (9), in promoters of many genes (10,11) and in recombination sites (12). They form various secondary structures with differing topologies that can positively or negatively affect replication, transcription or recombination [reviewed in (13)]. Secondary structures in DNA and quadruplexes specifically can be sensitively experimentally detected and characterized by circular dichroism (CD) spectroscopy (14,15).

There are some indications for the role of unusual DNA structures in the life cycle of retroviruses. In retroviruses, a G-quadruplex is probably involved in dimerization of the HIV-1 genomic RNA molecules inside virus particles (16). Moreover, HIV-1 nucleocapsid protein can unfold quadruplexes (17). In mammalian L1 non-LTR retrotransposons (LINE), the long PPT located in 3′UTR can form a triplex (18) or intrastrand quadruplex (19) *in vitro* depending on ionic conditions. To date, the presence of potential quadruplexes/triplexes has not been studied in LTR retrotransposons that are the most abundant elements in many genomes, e.g. in plants. Owing to the massive amount of genomic data that have been collected over the past decade, *in silico* analysis is now possible. Computational studies can improve our knowledge of sequence–structure relationships in biologically important genomic regions.

In this study, we analyzed 18 377 plant LTR retrotransposons and found that potential quadruplex-forming sequences (PQSs) are preferentially located inside LTRs at specific distances upstream as well as downstream of predicted promoters, whereas triplex-forming sequences (PTS) were most frequent in 5′ and 3′UTR.

## MATERIALS AND METHODS

### Sequence data

A collection of 88 730 predicted LTR retrotransposon sequences was used in the analysis. The sequences were obtained from publicly available genomes of 21 plants in a previous study described in [20]. These were subjected to analysis by LTR element annotation software and specialized programs for triplex and quadruplex DNA detection. A small subset of sequences with the most frequently occurring quadruplex pattern spacing (Table 1) was selected for experimental study.

**Table 1.** Oligonucleotides used in this study

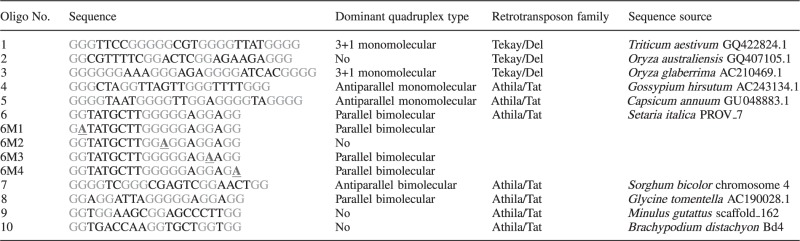

Names and the sequences of oligonucleotides are shown. The dominant type of quadruplex (if any) adopted by specific oligonucleotide is indicated. Groups of guanines are shown in orange. Nucleotides changed in the modified versions of oligonucleotide 6 are shown in underlined blue. Adenine was used as a replacement for guanine to see if quadruplex DNA would form with less guanines.

### Software and computational procedures

Computational analysis used a number of software packages and in-house scripts to carry out (i) LTR retroelement detection in genomic sequences and (ii) annotation of detected LTR elements and statistical analysis of the annotated features and underlying sequences.

#### LTR retroelement detection in genomic sequences

The presence of LTR retrotransposons and their exact positions were determined using the LTR Finder software (21). The program evaluates several properties indicative of the presence of LTR retroelement in an analyzed sequence, such as *gag* and *pol* domain similarity via pfscan profiles, duplication of LTR outside the *gag–pol* domains, primer-binding sequences, PPT presence and the exact nucleotides at important boundaries, such as 5′ and 3′ ends of the LTRs. The detected sequences were saved in FASTA format that identified the source genome/sequence and the position of the detected element in this sequence. As this procedure also returned fragments of retroelements, we included a filtering step in our analysis to work only with LTR retroelements containing a complete set of GAG, RT and INT domains after annotation. Sequences were also reverse-complemented whenever these domains were located on the minus strand of the analyzed genome. Finally, the remaining elements (∼25 000) were tested for the presence of direct repeats between multiple LTRs by BLAST, comparing the candidate full-length sequences against themselves. One quarter of the sequences did not pass this test and were removed from the analysis as nested sequences. The scripts used to carry out these steps are provided as Supplementary Material.

#### LTR retroelement annotation pipeline

Detected LTR elements were subjected to detailed annotation of the sequences by specialized in-house scripts written in *Perl* and *R*. These scripts (available as Supplementary Material) used FrameD++ (22) to find extra open reading frames (ORFs) in some of the retroelements. Triplex DNA-forming potential was evaluated using the *R*/Bioconductor triplex-1.0.8 program (23,24). This program not only detects nucleotide triples present in triplex DNA but also gives penalties for consecutive GCC triples and performs complete dynamic programming for treatment of inserts and deletions. PQSs were identified on both strands using a regular expression of the form 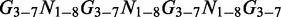 (PQS3). A modification of the pattern with only two obligatory Cs or Gs per cluster (PQS2) was used to map the background from which the GGG quadruplexes may have emerged. Highly likely promoter sequences were determined by the program Promoter 2.0 (25) with a quality cutoff of 0.9.

#### Randomization of sequence using a dinucleotide Markov model

To gauge the significance of identified PQSs, we compared the observed numbers of PQS sites with numbers expected from randomized LTR elements. The sequences were semi-randomized to follow the same dinucleotide sequence composition model as the original in a 150-bp window; however, a new sequence was generated to replace the original. This technique was used previously for identical purposes in humans and Arabidopsis (26,27). We wrote a dedicated *Perl* script mm.pl to carry out this operation. Downstream analysis was a subset of calculations carried out on the real sequence data.

#### CD spectroscopy and polyacrylamide gel electrophoresis

The lyophilized oligonucleotides were purchased from Generi Biotech (Hradec Kralove, Czech Republic) and dissolved in 1 mM sodium phosphate buffer with 0.3 mM EDTA, pH 7, to give a stock solution concentration of 100 OD ml^−1^. The precise sample concentrations were determined from the absorption measured at 90°C in the above buffer using molar absorption coefficients calculated according to (28). UV absorption spectra were measured on a UNICAM 5625 UV/VIS spectrometer. Before any measurements, the DNA samples were denatured for 3 min at 90°C. CD spectra were measured using a Jasco 815 dichrograph in 1 cm Hellma cells, placed in a thermostated holder. CD was expressed as the difference in the molar absorption of the right-handed and left-handed circularly polarized light, 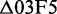, in units of one per mole and centimeter (M^−1 ^cm^−1^). The molarity (M) was related to nucleosides. Experimental conditions were changed directly in the cells by adding concentrated solutions of potassium chloride, and the final sample concentration was corrected for the volume increase. All the presented K+ dependences were measured at 20 and 1°C.

Native polyacrylamide gel electrophoresis (PAGE) was run in a temperature-controlled electrophoretic apparatus (SE-600; Hoefer Scientific). The gel concentration was 16% (29:1 monomer to bis ratio; Applichem). Two micrograms of oligonucleotide dissolved in 10-mM potassium phosphate and 135-mM potassium chloride was loaded on the gel and electrophoresed at 22°C for 18 h at 30 V. Gels were stained with Stains All (Sigma) and scanned using the Personal Densitometer SI, model 375-A (Molecular Dynamics).

## RESULTS

**Figure 1.**
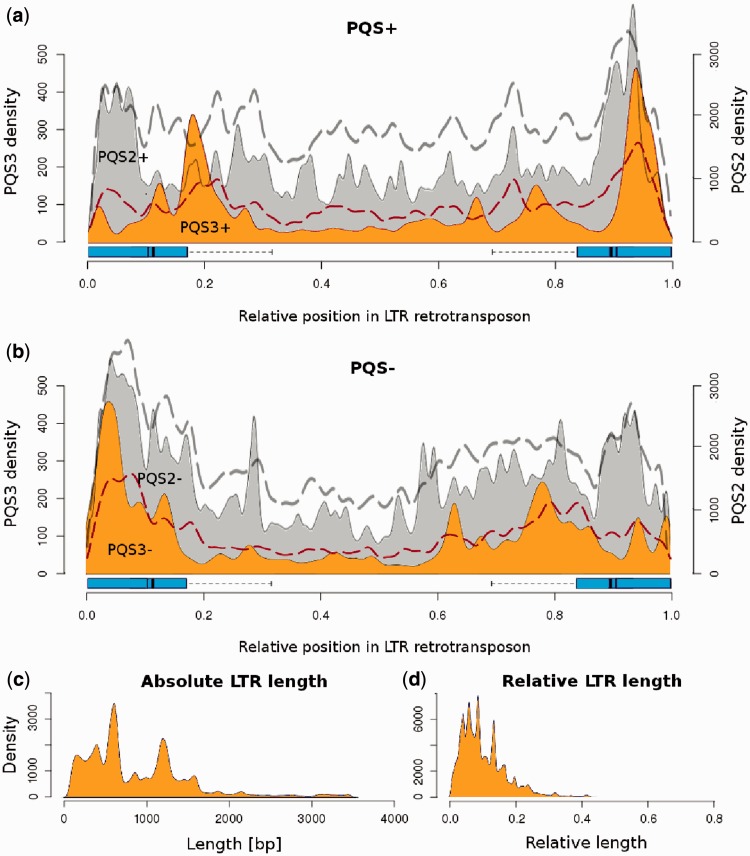
Occurrence of PQSs along LTR retrotransposons. The density of PQS clusters containing a minimum of four adequately spaced GGG groups (orange) in the (**a**) sense strand (PQS3+) and (**b**) antisense strand (PQS3−) visualized along elements. Dashed lines show expected density of PQS as predicted by a dinucleotide frequency Markov model applied as described in (26) (PQS2, upper line; PQS3, lower line). Sliding window covered 5% of the element length. LTRs are marked with a box extending to the third quartile (75% LTRs are shorter) and a one-sided error bar showing where the longest detected LTR begins (right LTR) or ends (left LTR). Dark bars represent the median (thin bar) and average (thick bar) LTR length. PQS2 clusters (gray) are shown at 6× lower density for comparison purposes (right axis). The length distribution of LTRs in base pairs (**c**) or relative length inside retrotransposons (**d**).

We collected 88 730 candidate elements from 21 plant genomes, yielding 18 377 full-length LTR retroelements after filtration. We searched their sequences for motifs that are known to adopt quadruplex or triplex structure. To search for PQSs, we used motifs containing four runs of three or more guanines separated by short stretches of any base: 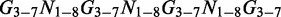. PQSs that contained four runs of at least three guanines in a single block (PQS3) were localized in discrete regions of TEs. We found that PQS3 present in plus strand (PQS3+) were frequently localized in the 3′ half of both left and right LTRs (Figure 1a and Supplementary Figure S1). On the other hand, PQS3 present in the minus strand (PQS3−) mapped predominantly to the left part of left LTR (Figure 1b, Supplementary Figure S1). The peak corresponding to the PQS3− was high in the left LTR, whereas it was blurred in the right LTR, partly because of different relative beginnings of the right LTRs (Figure 1b). This effect can be assessed by comparing the positions of PQS relative to element length with respective positions of PQS relative to LTRs (Supplementary Figure S1). We also searched for PQS2 containing runs of only two guanines, which do not readily form quadruplex (Figure 1a and b, gray). We found that PQS2 were more evenly distributed along entire LTR retrotransposons than PQS3, with marginal underrepresentation outside LTRs. This underrepresentation was more evident in PQS2+ than in PQS2− (Figure 1a and b). PQS2 sequences are known to form quadruplexes only under more restrictive conditions, such as lower temperature, higher potassium concentration or shorter loop length (29,30). We also evaluated the length of the LTRs of all LTR retrotransposons used in this study and found that the most frequent length was ∼600 bp and that the majority of LTRs did not exceed 2 kb (Figure 1c). Most LTRs represented maximally 20% of retrotransposon length each (Figure 1d).

To better gauge the significance of the detected PQS patterns, we compared the observed numbers of PQS sites with numbers expected from semi-randomized LTR elements (see ‘Materials and Methods’ section) (26,27). The expected frequency of PQS sites along the LTR elements is shown in Figure 1 by the dashed lines. PQS2 elements were within or below expectations, whereas PQS3 sites in both LTRs were present more often than expected.

PQS2 sequences were analyzed for loop lengths. The most frequent number of nucleotides between consecutive groups of two or more Gs/Cs was 1, 4 and 7 (data not shown). These numbers indicate a possible sequence periodicity of 3 for PQS2 clusters (as the period becomes 3, 6 and 9 after adding the two guanines). At an early stage of our study, we selected 10 PQSs from a small set of sequences carrying this specific pattern of spacing. Subsequently, we used them to design oligonucleotides (Table 1) and experimentally tested them for quadruplex formation.

**Figure 2.**
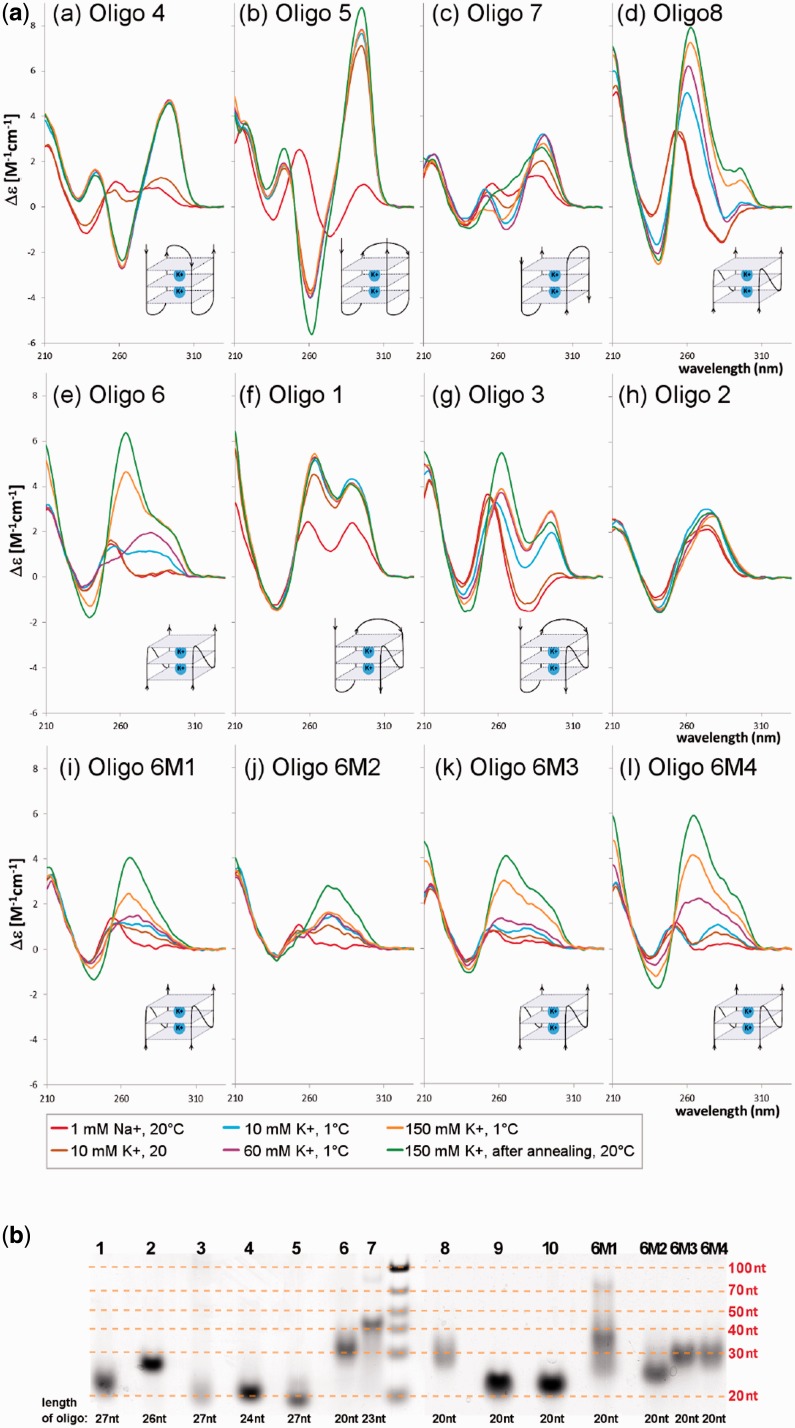
(**a**) CD spectra of the oligonucleotides shown in Table 1. Sketches correspond to the most probable folding of particular quadruplexes conforming to CD and electrophoretic results. The blue blocks depict quadruplex cores. (**b**) Native PAGE of oligonucleotides; each line is labeled by oligonucleotide number (Table 1).

To test conformational properties of the most common PQS present inside plant LTR retrotransposons, we measured CD spectra of 10 oligonucleotides corresponding to PQS containing four runs of two to four subsequent guanines (Table 1). The selected oligonucleotides were mostly unstructured in low salt solution and absence of potassium ions. On gradually increasing potassium concentration, most oligonucleotides adopted quadruplex structure. Oligonucleotides 4, 5 and 7 provided CD spectra with dominating positive band at 295 nm and a negative one ∼260 nm (Figure 1a), which is characteristic of antiparallel quadruplex (15). Oligonucleotides 6 and 8 preferentially adopted parallel-stranded quadruplex (Figure 2a) as indicated by CD spectra containing the high characteristic band at 260 nm (15,31). CD spectra of the oligonucleotides 1 and 3 contained two positive bands, ∼260 and 295 nm (Figure 2a), as commonly observed for so-called (3 + 1) quadruplex folding (15). While the former oligonucleotide formed a single probable (3 + 1) conformation, the latter one is rather a mixture of (3 + 1) and parallel quadruplexes. This is also indicated by the native PAGE, which shows the presence of heavier multi-molecular, probably parallel quadruplexes (Figure 2b). The native PAGE qualitatively indicates that three oligonucleotides formed intermolecular quadruplexes: bimolecular for oligonucleotides 6 and 8, while oligonucleotide 7 apparently also formed a minor population of four molecular quadruplexes. Quadruplexes of sequences 1, 4 and 5 were exclusively intramolecular. Only three oligonucleotides, 2 (Figure 2a), 9 and 10 (not shown), did not switch to quadruplex up to 150 mM K^+^, as exemplified by oligonucleotide 2 (Figure 2a). Some oligonucleotides (1,4,5) adopted quadruplex at only 10 mM K^+^, while others (3,6,7,8) transformed to quadruplex continuously with increasing K^+^ concentration. Low temperature (1°C) helped to stabilize quadruplex in most oligonucleotides, but better quadruplex stabilization was sometimes attained only by heating the oligonucleotides in 150 mM K^+^ to 90°C and slowly annealing them while decreasing the temperature to 20°C (Figure 2a, oligonucleotides 6, 6M1, 6M3 and 6M4). Sketches in Figure 2a correspond to the most probable quadruplex foldings based on CD and electrophoretic results. These data showed that oligonucleotide containing blocks of three or more guanines readily adopted quadruplex structures (oligonucleotides 1, 3 and 5) and the quadruplexes were intramolecular. The presence of less than three guanines in two or more blocks distinctly hindered quadruplex formation and only bimolecular quadruplexes were formed. This negative effect of only two guanines in blocks could be fended off by a long guanine block in the inner tetrad (oligonucleotides 8 and 6). In that case, the formed quadruplex was bimolecular and parallel. Oligonucleotides 2 (Figure 2a), 9 and 10 (not shown), containing only two guanines, did not switch to quadruplex.

The importance of the presence of the long guanine block follows from the mutated analogs of oligonucleotide 6, in which guanine at various positions was substituted by adenines. The substitution at the 5′-end GG block (oligonucleotide 6M1) destabilized the bimolecular quadruplex and gave rise to a smear of molecules including tetramolecular quadruplexes and also an unstructured fraction. The oligonucleotide substituted in the central part of the molecule (6M2), in which the 5-G block was destroyed, predominantly remained unstructured (which also follows from electrophoresis). On the other hand, the substitutions toward the 3′-end (6M3), and especially at the very end (6M4), did not obstruct quadruplex formation.

**Figure 3.**
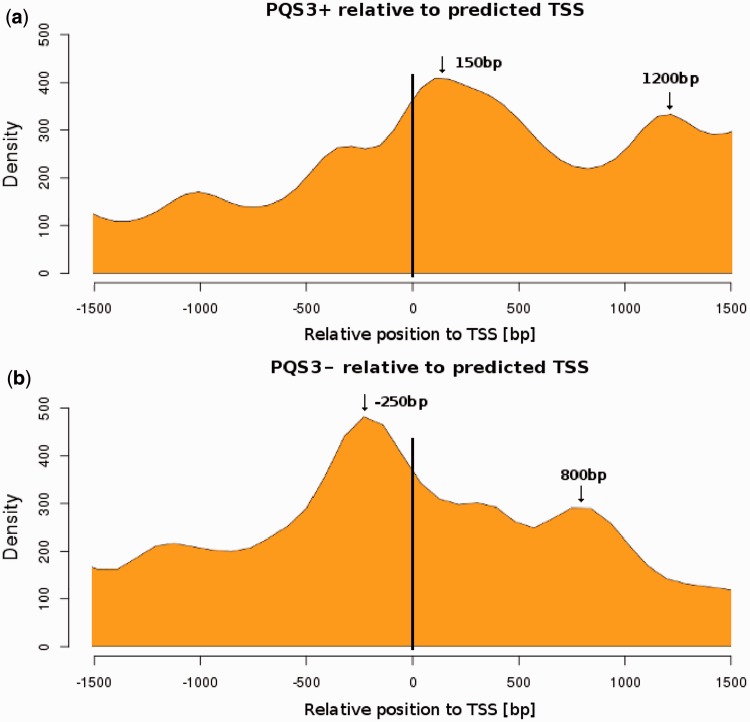
Localization of PQS in relation to predicted transcription start site (TSS). The density of PQS clusters (containing a minimum of 4 GGG runs) in LTR retrotransposons in the (**a**) sense orientation (PQS3+) and (**b**) antisense orientation (PQS3−) relative to the predicted TSS (negative values correspond to upstream position, positive values to downstream position). The preferential distances of PQS from TSS are marked by arrows.

Because quadruplexes can have regulatory effects on transcription, we searched for promoters and analyzed the relative location of PQS with respect to promoters. Promoters were predicted with Promoter 2.0 software, which showed they were localized inside LTRs. We found that PQS3+ were most frequently gathered ∼150 or 1200 bp downstream of promoters (Figure 3a), whereas PQS3− were most abundant ∼250 bp upstream of promoters but were also present ∼800 bp downstream of promoters (Figure 3b). When we analyzed data from individual species (data not shown), we found the presence and the exact position of the two peaks to be species dependent. For PQS3+, both peaks were only present in *Vitis*, *Sorghum* and *Zea*, whereas for PQS3*−*, this was the case in *Sorghum*, Eucalyptus and a pooled sample of the less represented species. The more prominent −250 bp PQS3− peak was absent in *Glycine*, *Mimulus* and *Zea*.

**Figure 4.**
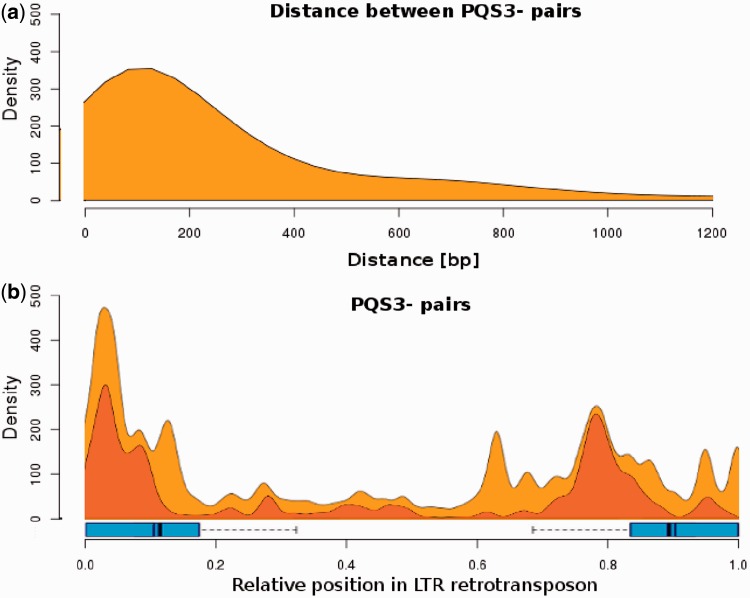
Occurrence of PQS double clusters. (**a**) The density of PQS clusters containing a minimum of four adequately spaced GGG groups against their mutual distance inside LTR retrotransposons. (**b**) The density of double PQS clusters (up to 1000 bp mutual intercluster distance) visualized along TEs (red), overlayed with PQS3− data from Figure 1 (orange).

We were interested if the presence of two or more peaks in the distribution of PQS in LTR retrotransposons (Figure 1a and b) as well as the presence of peaks at certain distances from promoters (Figure 3) was caused by the presence of doublets or triplets of PQS3−. For this reason, we measured the distance between neighboring PQS3− and found that PQS3− were often located within a distance of up to 400 bp (Figure 4a). Sixty-five percent of PQS3− sites were present as such doublets. When only doublets of PQS3− (localized up to 1000 bp apart) were visualized along the TEs, the accumulation in the right part of LTRs (downstream of promoter) was absent in contrast to single PQS3− (Figure 4b).

**Figure 5.**
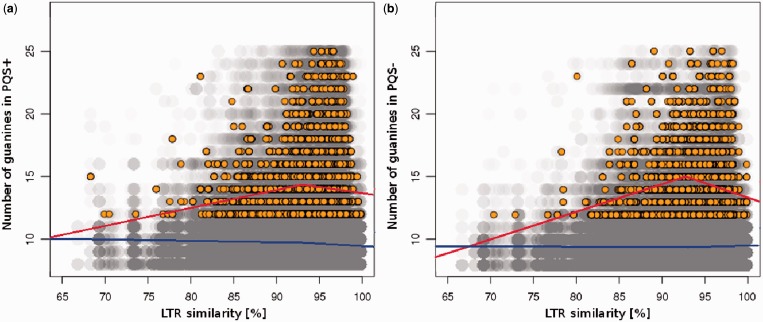
Quality of PQS clusters in relation to LTR retrotransposon age. The number of guanines presumed to form quadruplex plotted against LTR similarity (higher similarity corresponds to younger elements) of each LTR retrotransposon where a PQS cluster was present. Only PQS up to 25 guanines were considered. PQS3 (yellow circles) and PQS2 (gray circles) present in plus strand (left panel) or minus strand (right panel). Regression lines show the trends below and above LTR similarity of 93%. All PQS3 regressions (red) had slopes statistically different from 0 at the 0.01 significance level. Regression for PQS2 data is shown in blue.

If the PQSs have any biological role in the TE lifecycle, they should be found preferentially located in evolutionarily young LTR retrotransposons that were active recently. Therefore, we calculated the mutual divergence of LTRs of each TE (corresponding to their age) and also counted the total number of guanines in the corresponding quadruplex cluster (PQS score), skipping any guanines in presumed loops. As a cluster, we considered the four longest guanine runs separated by three loops. Lower guanine content could reflect quadruplex degeneration by point mutations. We found that long runs of guanines were present only in young LTR retrotransposons, whereas older LTR retrotransposons rarely contained long runs of guanines within their PQS (Figure 5). The dependence, however, was not uniform for elements of all ages. Linear regression analysis showed a two-phase trend of the dependence of PQS3 quality on age, where quality decreased with increasing age for LTR similarity <93% (Figure 5, red line, left) and quality increased with age for LTR similarity >93% (Figure 5, red line, right). No such trends could be found in PQS2 data (Figure 5, blue line).

**Figure 6.**
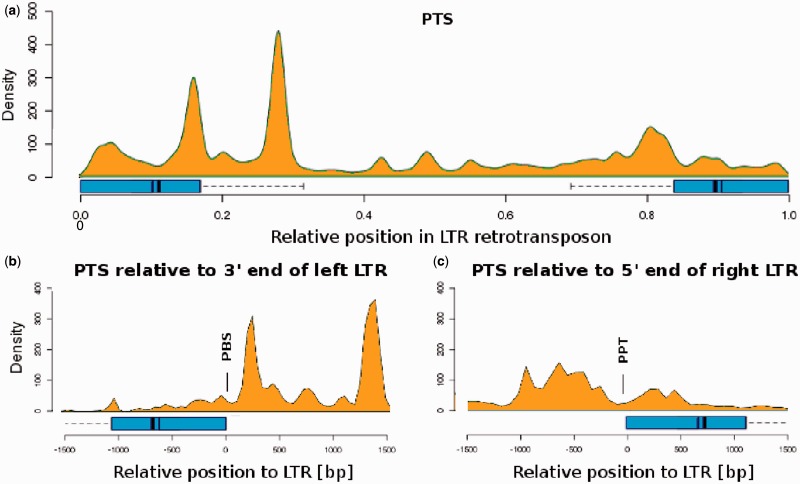
Occurrence of PTS along LTR retrotransposons. The density of detected PTSs visualized along elements (**a**). Sliding window covered 5% of the element length. LTRs are marked with a box extending to the third quartile (75% LTRs are shorter) and a one-sided error bar showing where the longest detected LTR begins (right LTR) or ends (left LTR). Dark bars represent the median (thin bar) and average (thick bar) LTR length. Localization of PTS relative to the 3′-end of the left LTR (**b**) and relative to the 5′-end of the right LTR (**c**) is shown.

Potential triplex sequences (PTS) that are characterized by polypurine/polypyrimidine runs had several distribution peaks along LTR retrotransposons but were lower in the central regions containing *gag* and *pol* genes and LTRs (Figure 6a). Similarly as in PQS, we related the localization of PTS to the 3′-end of the left LTR and the 5′-end of the right LTR (Figure 6b and c). This analysis showed that the greatest enrichment of PTS was downstream of the left LTRs, where PBS is found, and upstream of the right LTR, where PPT is located (Figure 6b and c).

**Figure 7.**
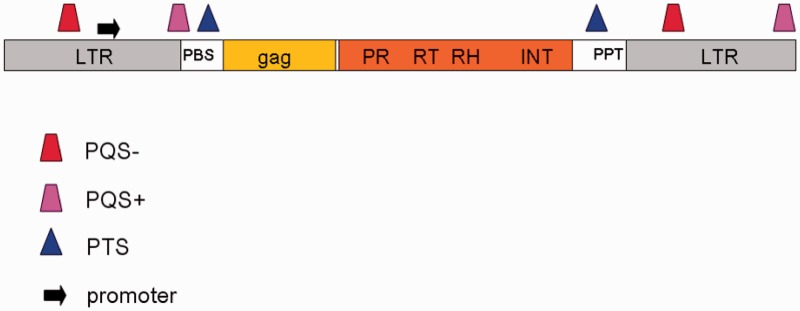
A schematic of PQS and PTS localization inside TEs. The most frequent localization of PQS, PTS and predicted promoters is marked together with *gag* and *pol* genes, PBS, PPT and LTRs.

Based on our results, we proposed a scheme of a typical plant LTR retrotransposon with indicated potential quadruplex- and triplex-forming regions (Figure 7). Taken together, quadruplexes and triplexes were found mostly in LTRs and other non–protein-coding regions separating basic structural/functional modules of LTR retrotransposons.

## DISCUSSION

In this study, we found marked enrichment of PQS and PTS motifs inside specific regions of plant LTR retrotransposons. Such gathering of these unusual DNA conformations could have a functional role in the life cycle of LTR retrotransposons or recombination-based reshuffling of plant genomes that are flooded with LTR retrotransposons.

Our results support the importance of subsequent guanine number in sequences forming quadruplexes. Their importance was underlined by the contrasting distributions of PQS3 and PQS2 in plant LTR retroelements, especially their proportions in LTRs (Figure 1). The importance of length of guanine runs is also evident from our analysis of the dependence of PQS quality (expressed as the number of guanines presumed to form quadruplex) on LTR retrotransposon age (Figure 5). Our experimental results support the bioinformatics analysis showing the importance of the number of guanines, where oligonucleotides with prevalence of guanine runs shorter than 3 Gs often failed to form monomolecular quadruplexes, or any quadruplexes at all (Table 1). The dependence of quadruplex formation on the length of guanine blocks and intervening loops has been studied on model sequences by a number of authors, including (32–37). Our study compares quadruplex formation in natural sequences containing a spacing pattern found to be abundant in plant retrotransposon G-rich sequences.

The abundance of PQS upstream and downstream of promoters indicates that quadruplexes may play a role in both initiation of transcription and elongation of RNA, respectively. The localization of quadruplex DNA upstream of promoters in the minus strand may stimulate transcription of retrotransposons by maintaining the transcribed region in a single-stranded conformation (13). Such explanation is consistent with our finding that recent and active LTR retrotransposons have more guanines in their PQS clusters, possibly a result of fixation of better quadruplexes during element evolution (Figure 5a). The localization of quadruplex DNA downstream of promoters in plus strand, in contrast, indicates that guanine quadruplexes are probably also formed in RNA transcripts. They may suppress elongation of the RNA strand or modulate its processing or interaction with proteins. This is in agreement with the lower number of guanines we found in PQS of old elements (Figure 5b). RNA quadruplexes are known to positively or negatively affect many processes in RNA biology like pre-mRNA processing, translation or RNA turnover and targeting (38). Surprisingly, the typical promoter–PQS distance function shown in Figure 4 had a second peak in both types of PQS, 1050 bp downstream of the peak closest to the promoter. We hypothesized that doublets of PQS were present at this distance, but analysis presented in Figure 5 showed the typical PQS–PQS distance to be 150 bp. This observation deserves future attention, as it could be connected to the existence of alternative PBSs (39) or alternative transcription start site (40), which however tend to be tens or hundreds bp apart, rather than a thousand.

Unusual DNA conformations might also be important for recognition of the transcripts by reverse transcriptase. It is consistent with our finding that RNA quadruplexes are often present at the 5′-end of left LTR (upstream of PBS) and 3′-end of right LTR (downstream of PPT) where they could interfere with extension of the first or second strand of cDNA during reverse transcription from PBS and PPT, respectively. On the other hand, our results showing underrepresentation of sequences forming unusual DNA structures in *gag* and *pol* genes of plant LTR retrotransposons are in agreement with significant repression of quadruplexes in the coding strand of exonic regions observed in human genome (41), probably because quadruplexes disfavor the formation of RNA. Our finding that PTSs are frequently localized inside 3′UTR demonstrates the potential of DNA triplexes to influence the synthesis of cDNA second strand (which starts in PPT) during reverse transcription.

Our results demonstrating the gathering of PQS and PTS in specific regulatory regions of plant LTR retrotransposons indicate that quadruplexes and triplexes, either as DNA or RNA molecules, could represent checkpoints of both transcription and reverse transcription. It is possible that quadruplexes and triplexes can also influence other stages of retrotransposons life cycle such as assembly of virus-like particles, dimerization or recombination of genomic RNA molecules. In this way, unusual DNA conformations can regulate life cycle of retrotransposons both in the nucleus and cytoplasm. Moreover, unusual DNA conformations can influence replication of host DNA (13) and may also play a role after retrotransposon insertions during recombination-based reshuffling of genomic DNA. Unusual DNA conformations are hot spots for recombination (13), and LTR retrotransposons are often the subject of ectopic recombination representing a unique mode of genome size reduction (42). The preferential location of PQSs and PTSs in LTRs or sequences directly flanking LTRs is intriguing. Apart from regulating transcription or retroviral RNA processing, these non-B DNA structures could participate in chromatin organization and methylation (43).

It has been previously shown that eukaryotic promoters are often enriched in PQS sequences (41). LTRs carry promoters; moreover, they are often the target of epigenetic regulation, whereas retrotransposons are methylated and inactivated by the host. Quadruplexes have been observed in unmethylated regions of eukaryotic genomes before (44,45). We speculate that the presence of quadruplexes in LTRs may be related to such inactivating mechanism, probably by interfering with the methylation process. Because quadruplexes formed on one strand would theoretically leave the other strand in a single-stranded state, it is possible they could hinder methylation of the surrounding sequences, even if they were rich in CpG and other methylable nucleotide pairs.

Another factor influencing DNA conformation is the ionic environment. Because quadruplexes are stabilized by potassium ions and triplexes by various mono- and divalent ions (46,47), the expression of LTR retrotransposons or genome reshuffling based on retrotransposons can sensitively respond to the cellular environment. This way unusual DNA conformations can regulate the activity of retrotransposons as well as modulate genome rearrangements in response to environmental challenges. An inherent part of such response is the reported activation of retrotransposons by environmental stress (48). This is especially evident in plants that cannot avoid adverse conditions by relocation. This is also the main reason why plant genomes are more dynamic than genomes of animals (49).

## SUPPLEMENTARY DATA

Supplementary Data are available at NAR Online.

## FUNDING

The Grant Agency of the Czech Republic [P205/12/0466, P305/10/0930, P501/10/0102, GA522/09/0083 and P501/10/P483], The Academy of Sciences of the Czech Republic [AV0Z50040702], by the project CEITEC–Central European Institute of Technology [CZ.1.05/1.1.00/02.0068] from European Regional Development Fund and by the project OPVK [CZ.1.07/2.3.00/20.0045]. Funding for open access charge: Research grants (to E.K. and M.V.).

*Conflict of interest statement*. None declared.

## Supplementary Material

Supplementary Data
