# Supplementary material for: Quadruplex-forming sequences occupy discrete regions inside plant LTR retrotransposons
Source: Nucleic Acids Res. 2013 Oct 6;42(2):968–78. doi: 10.1093/nar/gkt893 (PMC3902901; doi:10.1093/nar/gkt893)
Supplement: Supplementary Data [file supp_42_2_968__index.html]

Quadruplex-forming sequences occupy discrete regions inside plant LTR retrotransposons — Quadruplex-forming sequences occupy discrete regions inside plant LTR retrotransposons — Supplementary Data 

# Quadruplex-forming sequences occupy discrete regions inside plant LTR retrotransposons

## Supplementary Data

files

**Files in this Data Supplement:**

- Supplementary Data - zip file
