# Supplementary material for: Quadruplex-forming sequences occupy discrete regions inside plant LTR retrotransposons
Source: Nucleic Acids Res. 2013 Oct 6;42(2):968–78. doi: 10.1093/nar/gkt893 (PMC3902901; doi:10.1093/nar/gkt893)
Supplement: Supplementary Data [file supp_gkt893_suppl_data.zip › nar-01898-f-2013-File018.pdf]

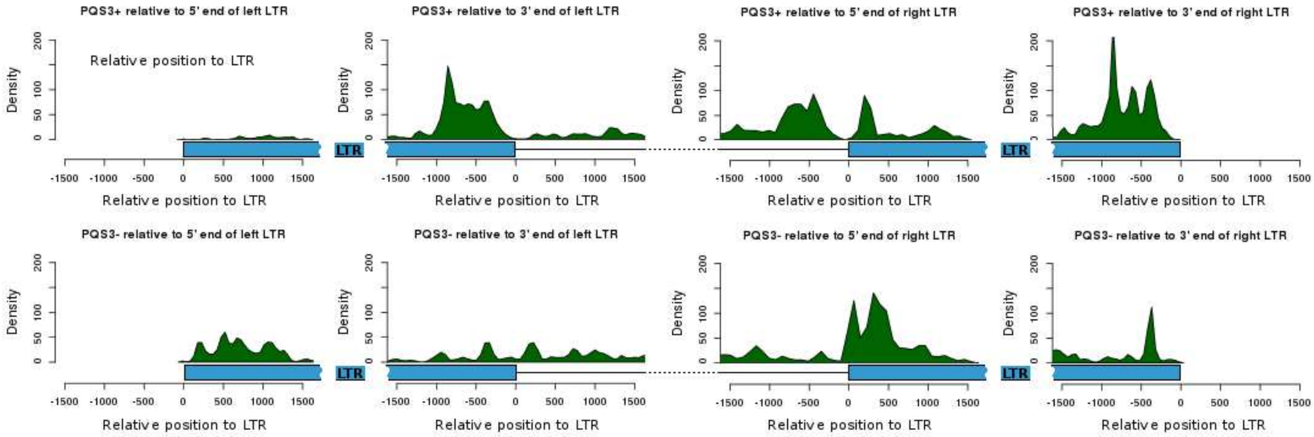

Figure S1. Localization of PQS in relation to ends of LTRs. The density of PQS3+ in relation to the 5' end and 3' end of the left and right LTR (top). The density of PQS3- in relation to the 5' end and 3' end of the left and right LTR (bottom). The position of LTRs in respect to the graphed data is shown as blue rectangles.
